# Liver-directed therapies for fibrolamellar carcinoma: A single-center experience

**DOI:** 10.32604/or.2024.052985

**Published:** 2024-11-13

**Authors:** SAM SON, AKSHAAR BRAHMBHATT, KEN ZHAO, BRETT MARINELLI, JAMES HARDING, WILLIAM JARNAGIN, GHASSAN K. ABOU-ALFA, HOOMAN YARMOHAMMADI

**Affiliations:** 1 Department of Radiology, Memorial Sloan Kettering Cancer Center and Weill Medical College at Cornell University, New York, NY 10065, USA; 2 Department of Medicine, Memorial Sloan Kettering Cancer Center and Weill Medical College at Cornell University, New York, NY 10065, USA; 3 Division of Hepatobiliary Surgery, Department of Surgery, Memorial Sloan Kettering Cancer Center and Weill Medical College at Cornell University, New York, NY 10065, USA

**Keywords:** Fibrolamellar carcinoma (FLC), Hepatocellular carcinoma (HCC), Liver-directed therapy (LDT), Transarterial radioembolization (TARE), Hepatic artery embolization

## Abstract

**Background:**

This article aims to present the single-institution outcomes of patients with Fibrolamellar Carcinoma (FLC) treated with liver-directed therapies (LDT).

**Methods:**

In this single-center retrospective study, all patients diagnosed with FLC who underwent LDT were identified. Between July 2012 and July 2023, six patients were identified. One patient was excluded due to bleeding. Demographic and clinical parameters were recorded. Complications within 30 days of the LDT were evaluated. Radiological treatment responses at 1, 6, and 12 months were assessed per mRECIST.

**Results:**

A total of five patients, which included three females and two males, were reviewed. Three patients were treated with transarterial hepatic embolization (TAE; n = 3), transarterial radioembolization (TARE; n = 1), and combined TAE + radiofrequency ablation (n = 1). The objective response rate at one month was 80% [CR = 2 (40%), PR = 2 (40%), and SD = 1 (20%)]. At 12 months (n = 4), two patients demonstrated CR (50%) and two demonstrated PR (50%). Overall survival from LDT at five years was 50%. There was no 30-day mortality among this group of patients or any adverse event attributable to the LDT.

**Conclusion:**

TAE, TARE, and ablation are safe and effective therapeutic options for FLC. Based on this study and previously published case reports, ablation and TARE yielded the most favorable results.

## Introduction

Fibrolamellar hepatocellular carcinoma, also known as Fibrolamellar Carcinoma (FLC), is a rare type of liver malignancy with an incidence of 0.02 per 100,000 [1]. It is responsible for approximately 1% of primary liver cancers and has a 1-year and 5-year cause-specific survival of 72% and 33%, respectively [2]. FLC is classified as a different disease entity separate from hepatocellular carcinoma (HCC) due to the differences in patient demographic and tumor characteristics. FLC generally affects younger individuals, with a median age of 22 years [3,4]. Alpha-fetoprotein, a common biomarker for HCC, is often negative in FLC. On histology, FLC is characterized by large tumor cells with eosinophilic cytoplasm surrounded by lamellated collagen fibers without cirrhosis [5]. Therefore, FLC diagnosis is predominately based on imaging and histopathological assessment [6]. Previous studies have demonstrated increased serum neurotensin in these patients and suggested that it might be a suitable tumor marker for diagnosing FLC and detecting recurrence after treatment [7]. Recent studies have suggested that the combination of tumor morphology and a unique functional chimeric protein, formed by the in-frame fusion of DNAJ homolog, subfamily B, member 1 (DNAJB1), and the catalytic subunit of protein kinase A (PRKACA) genes on chromosome 19, is thought to be specific to FLC and may play a role in its pathogenesis [6,8].

FLC presents most commonly as a large solitary lesion with a centrally located scar [9]. Complete resection is the standard of care in selected patients and may be curative, although recurrence is common [10,11]. However, there is no standard of care for unresectable FLC [12]. Additionally, FLC has a high rate of recurrence ranging from 33%–100% [13]. Available options are chemotherapy and locoregional liver-directed therapies (LDT), which include ablation, chemoembolization, transarterial hepatic embolization (TAE), and transarterial radioembolization (TARE). Unfortunately, due to the disease’s rarity, most published evidence are case reports [3]. Therefore, further evidence for effective alternative therapies that may guide clinical decision-making in patients ineligible for surgery is needed. This study aims to present the clinical outcomes of patients with FLC treated with LDT at our institution.

## Materials and Methods

This study was approved by the Institutional Review Board at Memorial Sloan Kettering Cancer Center (approval number: IRB# 16-402), and was in compliance with the Health Insurance Portability and Accountability Act. In this single-center retrospective study, all patients diagnosed with FLC who underwent LDT were identified. Between July 2012 and July 2023, six patients were identified. One patient was treated for bleeding and was excluded from the final review, resulting in five patients for further analysis. Demographic and clinical parameters, including age, gender, treatment, recurrence rate, and survival data, were recorded.

The modality of LDT used was based on the consensus of the institution’s multi-disciplinary board meeting. LDT was performed by three different interventional radiologists with more than ten years of experience. TAE was performed with 40–120-μm Tris-acryl gelatin microspheres (Embosphere Microspheres; Merit Medical, South Jordan, Utah) and/or 100 μm Poly-Vinyl Alcohol depending on the operator’s preference, as previously described [14]. One patient underwent TAE plus radiofrequency ablation (RFA; Covidien, Mansfield, MA, USA). One patient underwent microwave ablation (MWA; Ethicon, Raritan, NJ, USA). Finally, one patient underwent transarterial radioembolization (TARE) using glass microspheres (Thera Sphere; Boston Scientific/BTG, Marlborough, MA, USA). Dosimetry was performed using a medical internal radiation dosimetry model.

Complications within the first 30 days after the 1^st^ TAE or TARE that were directly related to the procedure were included. Cross-sectional imaging was reviewed by two board-certified attending interventional radiologists (K.Z. and H.Y., with 2 and 10 years of experience, respectively). Objective radiographic response (ORR) was defined as a combination of complete (CR) and partial response (PR). The response rate to the 1^st^ LDT was evaluated at 1, 6, and 12 months using the modified Response Evaluation Criteria in Solid Tumors (mRECIST) criteria [15].

## Results

A total of five patients, which included three females and two males, were reviewed. Three patients had positive molecular testing for DNAJB1-PRKACA fusion. The median age at diagnosis was 20.2 ± 5.3 years (range = 14.3–28.3). All patients had disease recurrence after previously performed curative-intent hepatic resection(s) and were referred for LDT for treatment of the recurrence. Three patients had additional resections for extrahepatic disease. Post-surgical resection recurrence-free interval was 28.9 ± 18.6 months. The average time from the date of surgery to LDT was 53.1 ± 55.6 months. There were no patients who presented with major preprocedural morbidity, including concomitant hepatic, cardiovascular, or metabolic conditions. Table 1 demonstrates the demographic characteristics of the patients along with surgical history and systemic treatments that the patients received before and after LDT.

**Table 1:** Demographic characteristics of patients

Patient number	Gender	Age at diagnosis (years)	Hepatic resections prior to LDT	Extrahepatic metastasis at the time of diagnosis	Systemic therapy prior to LDT	EOSP status	EOSP disease status	Survival from diagnosis (years)
1	F	28.3	Left hepatectomy and posterior lymphadenectomy	No	No	Alive	NED	11.3
2	M	20.2	Left hepatectomy and posterior lymphadenectomy, partial right hepatectomy	Yes	Yes	Alive	NED	14.9
3	F	23.3	Right trisegmentectomy	No	No	Deceased from clinical decline after recurrence	–	9.7
4	F	18.1	Left hepatectomy, posterior lymphadenectomy, and pancreatic and splenic resection with pancreatojejunostomy	Yes	No	Deceased from disease	–	3.2
5	M	14.3	Right hepatectomy and lymphadenectomy (peri-portal, peri-hepatic and celiac nodes)	No	Yes	Alive	SD	5.8

Note: LDT = Liver-directed therapy, EOSP = End of study period, NED = No evidence of disease.

Two patients had multifocal hepatic disease and three presented with solitary disease (Table 1). The two patients with multifocal disease were treated with TAE. Patient 1 was retreated with TAE plus MWA 2.5 years later for recurrence of disease and has remained disease-free since, for 5.2 years. All three patients presenting with solitary disease had recurrence along the prior surgical resection margin. One patient was treated with TAE plus RFA, one with TAE only, and the last patient was treated with TARE. Lesion characteristics and LDT performed are summarized in Table 2.

**Table 2:** Treatment and response

Patient number	Pattern	Largest lesion size prior to LDT	Initial LDT performed	Time to recurrence or progression from date of LDT (years)	1-month response	6-month response	12-month response
1	Multifocal	1.2 × 1.3 cm	TAE	2.4	PR	PR	PR
2	Solitary	2.8 × 2.0 cm	TAE + RFA	–	CR	CR	CR
3	Solitary	2.8 × 2.1 cm	TAE	2.3	CR	CR	CR
4	Multifocal	1.4 × 1.8 cm	TAE	–	PR	PR	PR
5	Solitary	4.3 × 3.4 cm	TARE	–	SD	–	–

Note: LDT = Liver-directed therapy, TAE = Transarterial Hepatic embolization, RFA = Radiofrequency ablation, TARE = Transarterial radioembolization.

Median follow-up after LDT was 3.8 years ± 2.5 years. The objective response rate at one month was 80% [CR = 2 (40%), PR = 2 (40%), and SD = 1 (20%)]. At 6 and 12 months (n = 4), two patients demonstrated CR (50%) and two demonstrated PR (50%) (Table 2). Of note, only four patients had response evaluation at 6 and 12 months due to one patient being lost to follow-up at approximately six months. Among the four remaining patients, overall survival (OS) from LDT at five years was 50%. There was no 30-day mortality or any adverse events attributable to LDT among this group of patients.

## Discussion

FLC is a rare, distinct primary liver tumor. It affects younger individuals with normal liver [16]. Additionally, these tumors possess a unique genomic profile, which differs from hepatoblastomas and conventional HCCs [1,17–19]. The tumorigenesis of FLC is thought to arise from a deletion of 400 kilobases on chromosome 19, which connects the DNAJB1 and PRKACA genes. Multiple reports have confirmed this finding, and the creation of this gene fusion in a CRIPR/CAS9 murine model has been shown to induce FLC-type tumors [6,17,20,21]. This fusion has also been found in other pancreaticobiliary malignancies [18].

FLC is generally treated with resection, given the usually large size at presentation, with both curative and cytoreductive intent, but has high recurrence rates ranging from 23%–60% [16,22]. Liver transplant is an option for patients with unresectable lesions without extrahepatic disease. A single review of 63 cases demonstrated that the OS after transplant for FL-HCC was 96%, 80%, and 48% at 1, 3, and 5 years, respectively [23].

Systemic therapy options for FLC include conventional therapies extrapolated from other primary tumors, but overall response rates are low and not durable [18]. Targeted agents have been tried, including bevacizumab, atezolizumab, and everolimus with and without estrogen deprivation therapy. However, these have failed to demonstrate any meaningful impact [24,25]. A single case report did demonstrate some response to erlotinib and bevacizumab treatment, but this was in a palliative setting [26]. One of the patients in the present study was treated with a targeted agent, ENMD-2076, as part of a clinical trial. This molecule is an aurora kinase inhibitor, which was hypothesized to target the overexpression of Aurora kinase A seen secondary to the DNAJB1-PRKACA fusion transcript. However, the trial demonstrated response in only one patient, with about half of the trial patients having stable disease [27]. There are some promising data on the use of immunotherapy in FLC with several ongoing trials (NCT04134559), (NCT04248569), and (NCT04380545); however, the data are still pending [24,28].

Interventional oncologic options can serve as a potentially curative treatment in cases of smaller newly diagnosed lesions, recurrence after resection, and/or provide locoregional control as a bridge to transplant. However, given the rarity of this disease, little has been reported in the literature on locoregional options. In the current review, three patients were treated with TAE. The patients remained disease-free for two years. One patient showed partial response to the treatment and later developed new lesions.

There is minimal data on endovascular liver-directed therapy for FLC and no known data on TAE. In a review of pediatric HCC treated by conventional transarterial chemoembolization (cTACE), one patient had FLC. This patient was initially treated with curative intent right hepatectomy but had a left-sided recurrence that was treated by cTACE using lipiodol, cisplatin, and doxorubicin. The patient showed minimal response and later underwent liver transplantation [29].

Several lesions in this present study were treated with ablation. Patient 2 and retreatment in patient 1 were performed with TAE + RFA and MWA ablation, respectively. Patient 2 showed no recurrence during the study period of 2.5 years. Patient 1 did undergo two sessions of MWA for two distinct lesions. One of these lesions recurred, requiring resection, but the second treated lesion showed a complete response with no recurrence during the study period. While the data is limited, these results suggest that ablation can provide a durable response and can be used for curative-intent treatments in specific cases similar to conventional HCC [30,31].

One patient in the current study was treated with TARE. FLC is radiosensitive, and one case report has described the treatment of FLC with TARE, which led to a decrease in tumor size from 350 to 20 cm^3^, ultimately allowing for resection [32–34]. This patient was initially treated with TACE using doxorubicin eluting beads and was still unresectable despite having a partial response by mRECIST criteria. Subsequently, the patient underwent TARE and received 3.45 GBq via the right hepatic artery and another 1.18 GBq via the left hepatic artery 15 and 17 months after DEB-TACE. The patient in the current series was similar to this published case, with a large tumor centered in the caudate lobe without a good surgical option. This lesion was treated with 200 Gy. The patient had a one-month follow-up, which demonstrated stable disease. After nine months, the patient showed progression of disease and was started on targeted therapy.

There is a handful of other FLC treated with TARE in the literature. A review of pediatric hepatic malignancy treated with TARE included three patients with FLC. One patient treated for pain palliation expired two months after treatment. The other two patients had stable disease despite multiple sessions of TARE ranging from 50–218 Gy to the tumor with survival after TARE of 17 and 20 months [35]. An older report also described the use of TARE for FLC in a pediatric patient for palliative intent. This patient had a large tumor encompassing the entire right lobe with portal venous thrombus. The patient received 155 Gy across the entire right lobe and another 169 Gy in a second session six months later. The patient had stable disease at seven months after the initial dose but had worsening extrahepatic disease. The patient survived 20 months after the initial TARE treatment despite several systemic therapies [36].

The goal of this study was to present cases of FLC treated with LDT and discuss the results in context with the currently available literature. This study has several limitations that affect its generalizability. The small size of the study cohort may not be representative of the patient population. Additionally, this is a retrospective study conducted at a single institution and reflects findings limited by geographical region, patient demographic, and institutional practices. Therefore, the results should be interpreted as a supplement to the current data on FLC available in the literature.

## Conclusion

In conclusion, our study demonstrates that LDT is a safe and effective therapeutic option for unresectable FLC. It is a highly tolerable, minimally invasive procedure that provides durable disease control in a patient population at high risk for disease recurrence. Few effective options established for the treatment of unresectable FLR remain an issue in this patient population, and the results of this study suggest that LDT should be considered a viable therapeutic option. Currently, LDTs are infrequently utilized, with most of them performed on patients who have failed multiple other therapies. Earlier referral for LDT after diagnosis for curative-intent treatment could be a benefit for these patients. Furthermore, LDT could potentially be effective as a neoadjuvant or adjuvant therapy to surgery, which could further expand the role of LDT and improve survival in this patient population. Future studies are warranted as there is limited evidence in the literature on the treatment of FLR with LDT.

## Data Availability

The datasets generated during and/or analyzed during the current study are available from the corresponding author upon reasonable request.
